# A proteomics analysis of neointima formation on decellularized vascular grafts reveals regenerative alterations in protein signature running head: Proteomics analysis of neointima formation

**DOI:** 10.3389/fbioe.2022.894956

**Published:** 2022-08-30

**Authors:** Chunyang Chen, Ting Lu, Zhongshi Wu, Xinlong Xie, Yalin Liu, Can Huang, Yuhong Liu

**Affiliations:** ^1^ Department of Cardiovascular surgery, Second Xiangya Hospital of Central South University, Changsha, China; ^2^ Engineering Laboratory of Hunan Province for Cardiovascular Biomaterials, Changsha, China

**Keywords:** neointima formation/hyperplasia, small-caliber blood vessel grafts, proteomics, protein adsorption, and inflammation, regenerable

## Abstract

**Background:** Neointima formation contributes to vascular grafts stenosis and thrombosis. It is a complex reaction that plays a significant role in the performance of vascular grafts. Despite its critical implications, little is known about the mechanisms underlying neointima formation. This study compares neointima proteome in different stages and plasma samples.

**Methods:** Heterogenous acellular native arteries were implanted as abdominal aortic interposition grafts in a rabbit model. Grafts were harvested at 0.5, 1, 4, 6, 7, 14, 21, and 28 days post-surgery for histological and proteomic analysis of the neointima.

**Results:** Histological examination showed a transformed morphological pattern and components, including serum proteins, inflammatory cells, and regenerative cells. Proteomics analysis of the neointima showed distinct characteristics after 14 days of implantation compared to early implantation. Early changes in the neointima samples were proteins involved in acute inflammation and thrombosis, followed by the accumulation of extracellular matrix (ECM) proteins. A total of 110 proteins were found to be differentially expressed in later samples of neointima compared to early controls. The enriched pathways were mainly protein digestion and adsorption, focal adhesion, PI3K-Akt signaling pathway, and ECM-receptor interaction in the late stage. All distributions of proteins in the neointima are different compared to plasma.

**Conclusion:** The biological processes of neointima formation at different stages identified with proteome found developmental characteristics of vascular structure on a decellularized small vascular graft, and significant differences were identified by proteomics in the neointima of early-stage and late-stage after implantation. In the acute unstable phase, the loose and uniform neointima was mainly composed of plasma proteins and inflammatory cells. However, in the relatively stable later stage, the most notable results were an up-regulation of ECM components. The present study demonstrates an interaction between biological matter and vascular graft, provides insights into biological process changes of neointima and facilitates the construction of a functional bioengineered small vascular graft for future clinical applications.

## Introduction

Heart disease, especially coronary artery disease, is one of the leading causes of death worldwide (Tsao et al., 2022). Traditional treatments for coronary artery disease include coronary artery bypass grafting (CABG) and percutaneous coronary intervention (PCI). Vascular repair grafts are in high demand in the clinic. Over 400,000 CABG surgeries are performed in the United States, while 45,000 procedures are conducted in China annually (Mozaffarian et al., 2016) (Ma et al., 2020; Virani et al., 2020). For the time being, autologous grafts are the preferred material for vascular replacement; nevertheless, the ability to harvest arteries from the same patient is sometimes limited due to the patient’s pathophysiological state (not available in 25–30% of patients) (Faries et al., 2000). Consequently, synthetic vascular grafts (ePTFE (expanded polytetrafluoroethylene) and Dacron (polyethylene terephthalate)) have been implanted in millions of patients over decades.

However, these operations (CABG, PCI) often lead to the formation of sub-endothelial scars in the blood vessels called neointima. The formation of neointima can become a serious problem when it substantially reduces blood flow in the presence of stenosis. Similarly, heterogenous or synthetic tissue-engineered vascular grafts (TEVGs), are more prone to trigger thrombosis and neointima formation (Gong et al., 2016; Li et al., 2016) (Herrmann et al., 2019; Kirkton et al., 2019).

In coronary artery bypass graft and vascular injury models, neointima formation (NF) has been widely investigated. Neointima development is induced by various stimuli. Platelet activation, leukocyte recruitment, activation of the coagulation cascade (hours to days), smooth muscle cell (SMC) migration, and vascular remodeling (weeks to months) are all included in this 5-step summary (Collins et al., 2012) (Davies and Hagen, 1995) (Muto et al., 2010). It is commonly acknowledged that an excessive accumulation of myofibroblasts and SMCs plays a key role in the formation of the neointima (Ross and Glomset, 1973) (Roy-Chaudhury et al., 2009) (Lacolley et al., 2012).

However, in small caliber TEVG implantation models, the mechanisms of NF remain unknown. And since TEVG lacks endothelial cells and SMCs, blood contact causes plasma proteins and blood cells to adhere, and that is what facilitates neointima formation. Furthermore, the underlying mechanisms may alter in TEVG replacement models compared with the vascular injury models due to the absence of the SMCs in the vascular wall. Subsequent protein and cellular changes in each stage are mutually influenced (adhesion, aggregation, and proliferation of cells in each stage), and differences in cellular fate can affect the process. We have demonstrated that modification of the vascular material can influence the development of the neointima and that our previous protein analysis of the neointima after 4 weeks of *in vivo* implantation reveals the presence of both plasma proteins and regenerated proteins (Liu et al., 2022). Recent evidence suggests that the whole process may involve the interactions between serum proteins, platelets, inflammatory proteins/cells, fibroblast/SMC-like cells, and endothelial cells leading to progressive narrowing of the lumen (Watase et al., 1992) (Reinhardt et al., 2019).

A better understanding of neointima formation is of great clinical importance. To date, neointimal proteomics studies have been performed on femoral artery injury models (Wierer et al., 2021), and porcine coronary stent implantation models (Suna et al., 2018). In this model, existing research recognizes the critical role in neointima formation played by the proteins annotated for regulatory functions in cell migration, such as transient receptor potential canonical 6 (Trpc6) protein (Wierer et al., 2021), aggrecan (Suna et al., 2018), metalloprotease 22 (Zhang et al., 2019), β-Catenin (Riascos-Bernal et al., 2017), P-Selectin (Hayashi et al., 2000) and so on. However, these results were based on the vascular injury or in-stent model and it is unclear if these findings are applied to the TEVG replacement model in the absence of the SMCs in TEVGs.

For now, there are no studies on the time-resolved proteomic changes of neointima after TEVG replacement. It is critical to characterize the different stages of neointima formation. This study aims to unravel some of the mysteries surrounding the early neointima formation after the small-diameter TEVG implantation. Therefore, we established TEVG manufacturing, tissue collection, mass spectrometry, histological profiling, and statistics and bioinformatics to describe the proteome neointima following TEVG implantation and identify those proteins, networks, and cells most strongly associated with early neointima development. Such findings offer the opportunity to inform the design of TEVGs by specific modification, thus supporting viable neo-vessel formation without aggressive stenosis.

## Materials and methods

### Vascular graft preparation

**FIGURE 1:**
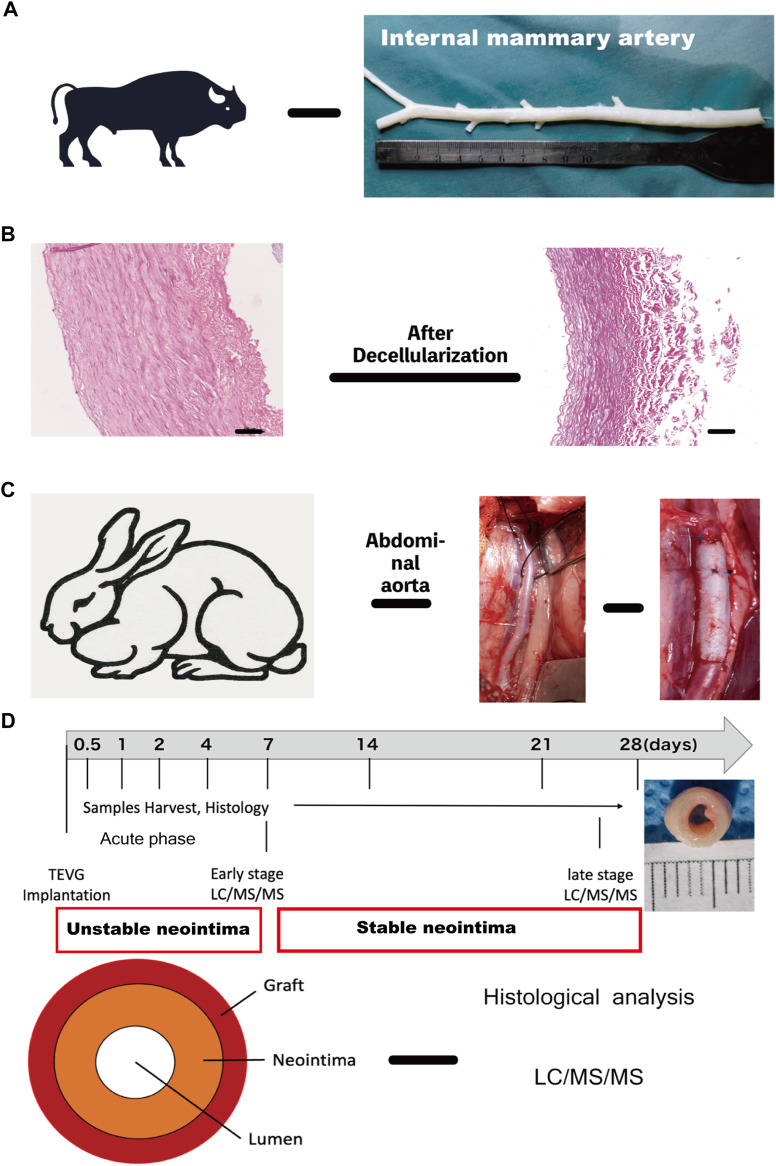
Experimental design and workflow for the proteomic and histological analysis **(A)** Internal mammary artery **(B)** H&E images of Native internal mammary artery and decellularized artery, scale bar = 100 μm. **(C)** Intraoperative image of TEVG implantation in the rabbit abdominal aorta. **(D)** Schematic overview of study design and neointima structure: grafts were implanted and harvested at 0.5, 1, 2, 4, 7, 14, 21 and 28 days for histological and proteomic analysis. (I: acute phase-unstable neointima: 0.5–4 days; II&III: early (1 week) and late-stage phase (3–4 weeks))TEVG, tissue-engineered vascular graft. TEVG: Tissue-engineered vascular graft; H&E: Haematoxylin and eosin stain.

Fresh bovine internal mammary artery (BIMA) was obtained from a rural slaughterhouse and was thoroughly cleansed of adhering tissues and residual fat before being rinsed with phosphate-buffered saline (PBS). Following that, 10 cm segments of BIMA were cut and kept at 4°C in PBS supplemented with 1 percent streptomycin/penicillin. Thereafter, BIMA was further decellularized as indicated in our flow scheme Figure 1A. Our decellularization procedure was based on a combination of physical, chemical, and biological methods and a system that consisted of an ultrasonic cell breaker, peristaltic pump, and a self-designed decellularized container. The procedure was as follows: First, we perfused BIMA for 24 h with 0.5% Sodium Dodecyl Sulphate (SDS, Sigma, L3771, United States) and 0.5% Triton X-100 (Sigma, X-100, United States), followed by a 12-h sonication treatment (20kHz, 200w). Second, grafts were rinsed with PBS solution using a peristaltic pump for 30 min before being put in a decellularized container with DNAse (40U/ml, Servicebio, G5043, China) and RNAse (0.3 mg/ml, Servicebio, G3413, China) for 24 h at 37°C in a water bath. Third, samples were cleaned in a perfusion system with PBS for 48 h following ribozyme treatment. In brief, the grafts were immersed in a cold PBS containing glucose (pH 7.6, Osm: 680 Osm) for 4 h followed by a 2-step crosslinking process. First, the grafts were incubated in a crosslinking solution, a cold PBS containing glucose (pH 7.6, Osm: 320 Osm) that comprised 0.1% methylene blue (Sigma, M9140, United States) for 4 h at 4–20°C in the dark. In the second step, a 500 W broad-spectrum light is used to react the crosslinking solution and DC graft for 48 h at 0–15°C with 10 ml/min air sparging through the stirring reaction solution. The grafts’ inner and outer walls were photo-oxidized for 24 h, with the reaction solution changing every 24 h. Finally, all of the grafts were rinsed in sterile PBS and kept at 4°C for further study in sterilized PBS. The image of the grafts is shown in Figure 1B. (Detailed data were in additional file 6. Supplementary Figure S1). The decellularization and dye-mediated photooxidation procedures were modified from our previous research (Lu et al., 2009) (Lu et al., 2010).

### Animal model, samples acquisition

The animal procedure was carried out by the Second Xiangya Hospital’s Department of Cardiac Surgery. 24 healthy male (*n* = 12) and female (*n* = 12) New Zealand white rabbits (2.5–3.0 kg) underwent abdominal aorta replacement (Figure 1C). Rabbits were given 30 mg/kg of sodium pentobarbital to anesthetize and sedate them. The aorta was dissected free of the surrounding tissues and inferior vena cava following the left paraspinal incision. Before implantation, intravenous heparin (1 mg/kg) was given, and end-to-end anastomoses with 7–0 prolene sutures were used to implant grafts (2 cm length/3.0 μm inner diameter) into the infrarenal aorta. To prevent the risk of graft thrombosis, rabbits received a dose of nadroparin intravenously 24 h after surgery and remained on this antiplatelet therapy until 1 week. All experiments were approved by the Institutional Animal Care and Use Committee (The Second Xiangya Hospital, Central South University, China) and in accordance with the Guide for Care and Use of Laboratory Animals.

The rabbits were divided into the following eight groups depending upon the interval of the observation period: Day-0.5 (*n* = 3), Day-1 (*n* = 3), Day-4 (*n* = 3), Day -7 (*n* = 3), Day-14 (*n* = 3), Day-21 (*n* = 3) and Day-28 (*n* = 3). The rabbits were killed at 0.5, 1, 2, 4, 7, 14, 21, and 28 days respectively after the operation. The harvested samples (each group) were divided longitudinally into two sections, one for light microscopic (LM) analysis, and another for proteomics (Figure 1D).

### Histochemistry and image analysis

The fixed, paraffin-embedded tissues were sectioned into 5 μm slices and stained with hematoxylin and eosin (H&E, Servicebio, G1005, China), Masson’s trichrome (Servicebio, G1006, China), elastic van-Gieson (EVG, Servicebio, GP1035, China), and Picro-Sirius Red staining (Abcam, ab150681, United Kingdom) according to standard protocols for histological evaluation. For immunohistochemical analysis, sections were dewaxed and rehydrated using xylene followed by a decreasing series of ethanol. Endogenous peroxidases were inhibited by incubating the slides for 15 min at room temperature in a 0.6 percent methanol-hydrogen peroxide solution. Unspecific binding sites were inhibited by incubating the slides for 30 min at room temperature in 1.5% normal goat serum. Primary antibodies were diluted in PBS and incubated overnight at 4°C. Primary antibodies were CD68 (1:200, Servicebio, GB14043, China), CD163 (1:25, Abcam, ab111250, United Kingdom), CD31 (1:200, Abcam, ab9498, United Kingdom), vWF (1:30, Abcam, ab778, United Kingdom), alpha-smooth muscle cell actin (1:200, Gene Tex, GTX18147, United States), vimentin (1:200, Abcam, Ab8978, United Kingdom), and calponin (1:500, Abcam, ab700, United Kingdom). Secondary antibodies (KIT-9701, Maixin Biotech, China) were applied for 1 h, followed by wash steps in PBS. DAB/AEC-Solution was used to detect positive signals. Sections were counterstained using Hematoxylin and dehydrated using a graded series of ethanol and xylene (AEC-solution cannot dehydrate using alcohol).

Image analysis was performed using ImageJ. Polarized light was used to image Picro-Sirius Red staining. Neointima thickness and area were quantified from 20X field images. The neointima area quantified the whole neointima, and each scan took 8 points all around the lumen circle. These data were averaged further over *n* = 3 rabbits to determine overall mean thickness and area ±SD at 0.5, 1, 2, 4, 7, 14, 21, and 28 days, hence yielding 24 data points per observation neointima for assessing neointima thickness changes.

### Proteins analysis after extraction from graft materials

In total, samples were analyzed by liquid chromatography-tandem mass spectrometry (LC-MS/MS) for the neointima (*n* = 3) blood plasma and *n* = 3 at each time point 0.5, 1, 2, 4, 7, 14, 21, and 28 days; We mixed the specimens (*n* = 3) at each time point in the acute phase due to the low mass of the protein specimens (particularly at day 0.5 to day 2) and ran a mass spectrometry analysis as the average expression; *n* = 3, early phase [day 7] and *n* = 6 late phase (day 21 to day 28).

The grafts’ neointima was dissected, washed, and wiped dry using dust-free paper. To solubilize protein, 8M urea +100 mM Tris-HCl (pH 7.2) lysate (with protease inhibitor) was added. The BCA method was used to determine the protein concentration. 500 g protein was isolated from each set of samples (basic near the mid-region), then digested overnight at 37°C with mass spectroscopy-grade Trypsin (V528A, Promega) at a ratio of 1:50. For mass spectrometry analysis, the peptide-containing solution was freeze-drained and the peptide was dissolved. For mass spectrometry analysis, the Nano LC-LTQ/Orbitraps MS was employed. Positive ion mode detection electrospray ion source with 2.4 kV spray voltage and 350°C ion source temperature. The first-level FT scan had a resolution of 60000FWHM, while the second-level IT scan had a resolution of 7500FWHM and collision energy of 35 percent in TOP20 mode. The MS RAW files in the Uniprot TrEMBL database were searched using the Thermo Fisher program Proteome Discovery 2.0.

### Computational mass spectrometry-data analysis and bioinformatic analysis

Mass spectrometry-data (MS RAW files) analysis was performed using MaxQuant software (Cox and Mann, 2008) (Delius et al., 2017) (version 1.6.15.0). For peptide and protein identification raw files were searched against the rabbit Uniprot FASTA database [UP000001811. fasta (v2021.03) (Swiss Prot and TrEMBL, Swiss Institute of Bioinformatics, Geneva, Switzerland, http://ca.expasy.org/sprot/)]. Details are seen in the additional file. (Additional file 6. MaxQuant setting parameters) Quantification of identified protein intensities was normalized to obtain label-free quantitation (LFQ) intensities (Cox et al., 2014). Feature matching between raw files was enabled. Further bioinformatic analyses were performed with DAVID software ([https://david.ncifcrf.gov] (https://david.ncifcrf.gov)). The Gene Ontology (GO) enrichment analysis and the Kyoto Encyclopedia of Genes and Genomes (KEGG) pathway database were used to annotate and classify the pathways of expressed proteins. The proteins of significantly regulated were input into the STRING program ([http://string-db.org/] (http://string-db.org/)) to identify potential protein-protein interactions (PPI) (Szklarczyk et al., 2015). Only interactions with a STRING combined score >0.7 were considered high confidence levels.

### Statistical analysis

Data were analyzed using the unpaired Student t test for differences between early and late stages of neointima. *p*-values were adjusted for multiple comparisons by the Benjamini–Hochberg method (Glickman et al., 2014). For component analysis, missing values (in the LFQ data) were imputed with a constant (30,000) close to half the smallest LFQ intensity. For all statistical comparisons, *p*-value < 0.05 regarded significant. The abundance ratio of a protein in the early-neointima compared to the corresponding later sample was considered to be significantly depleted in the neointima for a fold change (fc) < 0.5 or significantly enriched for fc > 2.0, and an adjusted *p*-value <0.1. All statistical analyses were performed using Perseus (Tyanova et al., 2016) (v. 1.6.15.0).

## Results

### Histology

**FIGURE 2:**
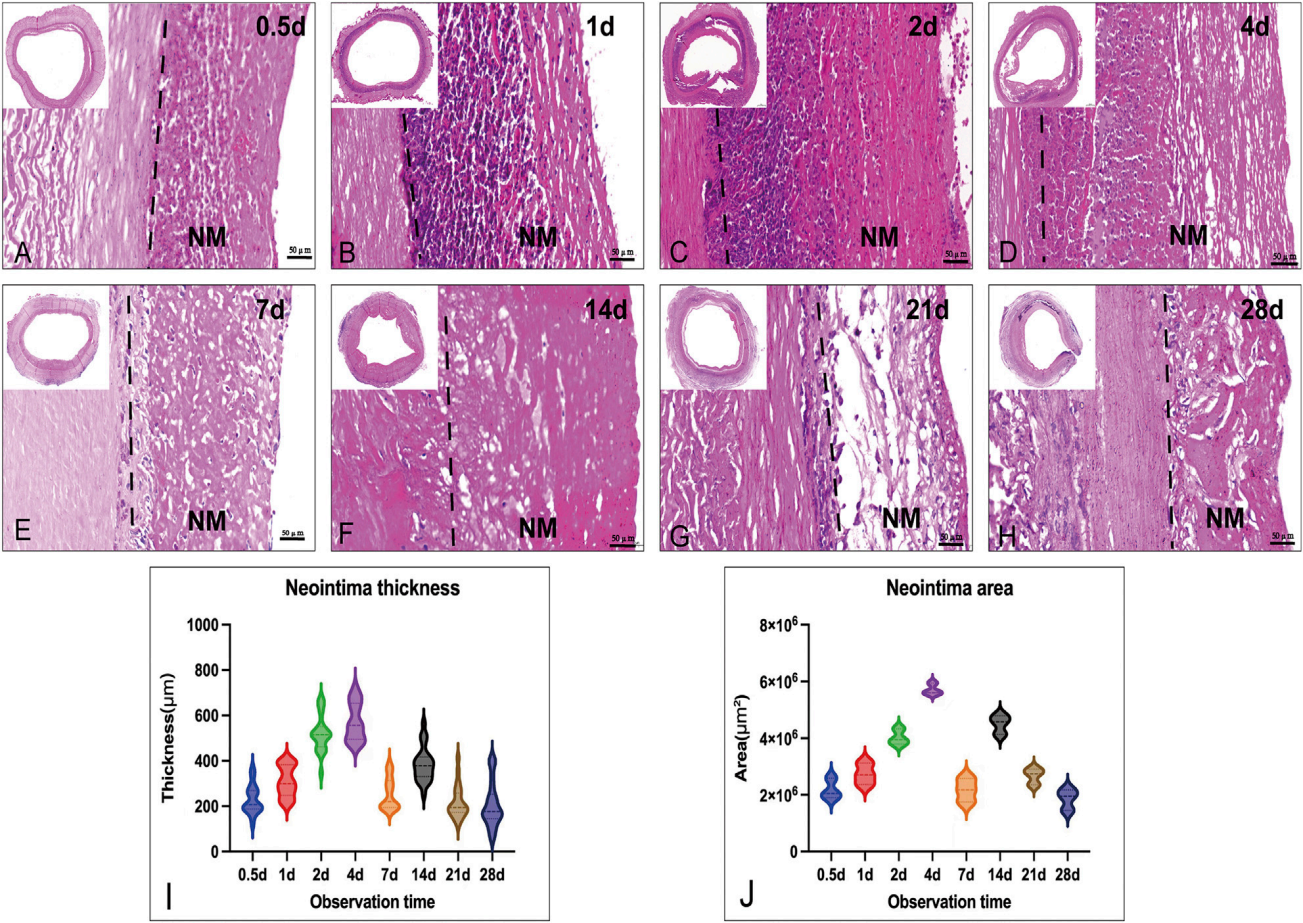
Histological images (H&E) of the neointima change in thickness and area in the vascular lumen at different times. **(A–H)**: the cross-section morphology of Implanted graft at different times (up-right: time points, scale bar = 50 μm); **(I)**: Measurements of average neointima thickness in different grafts; **(J)**: Measurements of the area in different grafts: Both thickness and area had the larger value at day 4 (thickness: 700 μm; area: 6 × 10 μm^2). NM: neointima.

Microscopic and immunohistochemical examination of the lumen area revealed a uniform pattern of the vascular graft with distinct features compared with the control decellularized graft. The neointima was between 80 um to 700 um (normal <50 um) thick with abundant serum proteins, ECM, and cells (Figure 2) (Westerband et al., 2000). Interestingly, the results indicated that a certain thickness of neointima formed on the lumen a few hours after implantation, with the first peak of maximum area and thickness around day 4 and the second peak at around 2 weeks.

### Temporal evaluation of cells aggregation and matrix remodeling by histological stain

**FIGURE 3:**
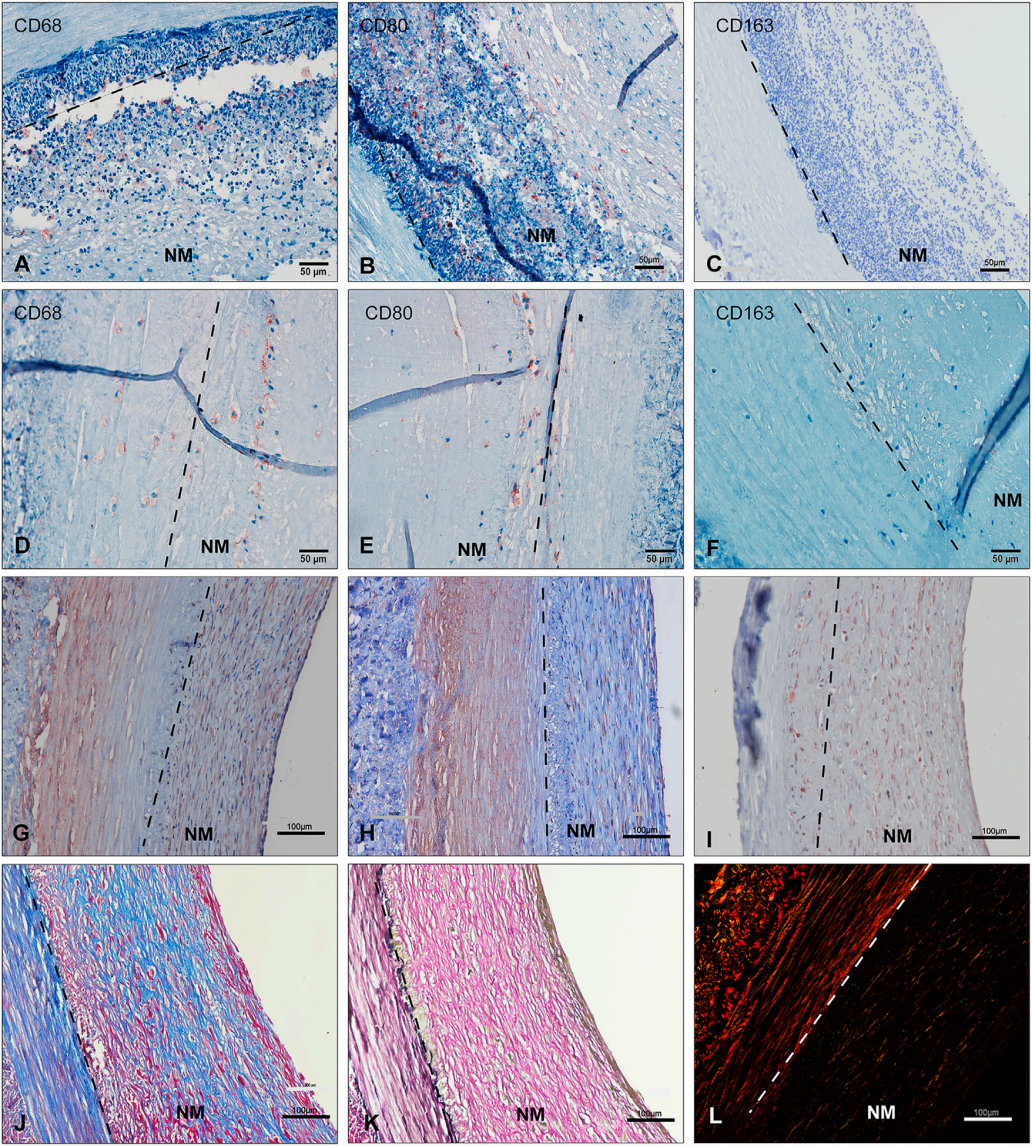
Immunohistochemical studies of neointima over time **(A–I)** EVG**(K)**, Masson**(J)** and Picro-Sirius Red staining(L) show the neointima to be rich in ECM proteins. Two types of cells are seen, either SMC-like cell for Alpha SMA**(G)**, vimentin**(I)**, and calponin**(H)** positive or CD68 **(A,D)**/CD80**(B,E)**-positive macrophages with CD163**(C,F)**-negative. **(A–C)**: unstable neointima --- before day 7; **(D–L)**: stable neointima --- day 21–28) (scale bar = 100 μm). NM: neointima region; ECM: extracellular matrix.

Routine H&E staining of graft cross-sections before and after implantation revealed *in vivo* neointima tissue generation and remodeling. Neointima tissue was defined as a layer in the lumen based on the graft (Figures 2A–H). As shown by H&E staining, the neointima thickness was dramatically increased (up to 700 μm) at each observation point in the early phase (first week). Due to the neointima formation, lumen loss was apparent. In the acute unstable phase (before 7 days), the major constituents of neointima were adsorbed proteins and acute inflammatory cells (mainly leukocytes: neutrophils), and the peak time was in 1 week (4–6 days). Interestingly, neutrophils faded in the first week and mainly proteins remained in the second week. After neutrophils disappeared, the neointima wall thickness was reduced and the structure was stable. During the late stage (3–4 weeks), the thickness of neointima was decreased to an average of 200 μm by the time observation ended (Figure 2I). At the 4th week after implantation, the graft lumen was covered with a layer of neo-generated tissue that was mainly composed of blue collagen fibers through Masson’s stain (Figure 3). Furthermore, we conducted a Picro-Sirius Red stain to confirm the components in the neointima (Figure 3).

### Immunohistochemical evaluation of inflammatory cells in neointima

Immunohistochemical staining showed that the majority of the host cells gathering on the lumen throughout all explant time points appeared to be non-immune and inflammatory cells. Neutrophils are the first inflammatory cells in the foreign body reaction. (Anderson et al., 2008). Macrophages and fibroblasts play important roles in inflammation and tissue remodeling (Wynn and Vannella, 2016). Specifically, lots of inflammatory cells were found in retrieved explant samples during the first week (most Ly6G+ and MPO+, additional file 6: 4. Supplementary Figure S3 MPO and LY6G). The staining revealed no CD163+ anti-inflammatory M2 macrophages in grafts at 4-week’s time point. Few CD80^+^ and CD68^+^ pro-inflammatory macrophages were identified in explants after 1 week of implantation. In some explants, several fibrosis bodies or giant cells were identified.

### Repopulation and evolution of SMC-like cells (alpha SMA+) and fibroblasts (vimentin+) within the neointima

Immunohistochemical staining for alpha SMA and vimentin revealed lots of regenerative cells within the late stage of the neointima layer. The alpha SMA + or vimentin + cells, likely smooth muscle cells (partially calponin+) or myofibroblasts, increased in density in the neointima tissue in the late implantation stage. These cells were correlated with new collagen fibers and became more elongated and aligned Figures 3J, K.

## Proteomics

### Overview of the proteome information

The implantation of all kinds TEVGs evoked a foreign body reaction in which a substantial number of plasma proteins attach and accumulate on the vessel’s luminal surface (and subsequently blood cells, mainly acute inflammatory cells), resulting in the creation of a neointima. The remodeling process of the neointima is partially affected by changes in the proteome of the neointima. We implanted decellularized BIMA into rabbits’ abdominal descending aorta and performed histological study [Figures 2, 3] and proteomics analysis at eight-time points: 0.5, 1, 2, 4, 7, 14, 21, and, 28 days following graft implantation to learn more about this process at the protein level.

**FIGURE 4:**
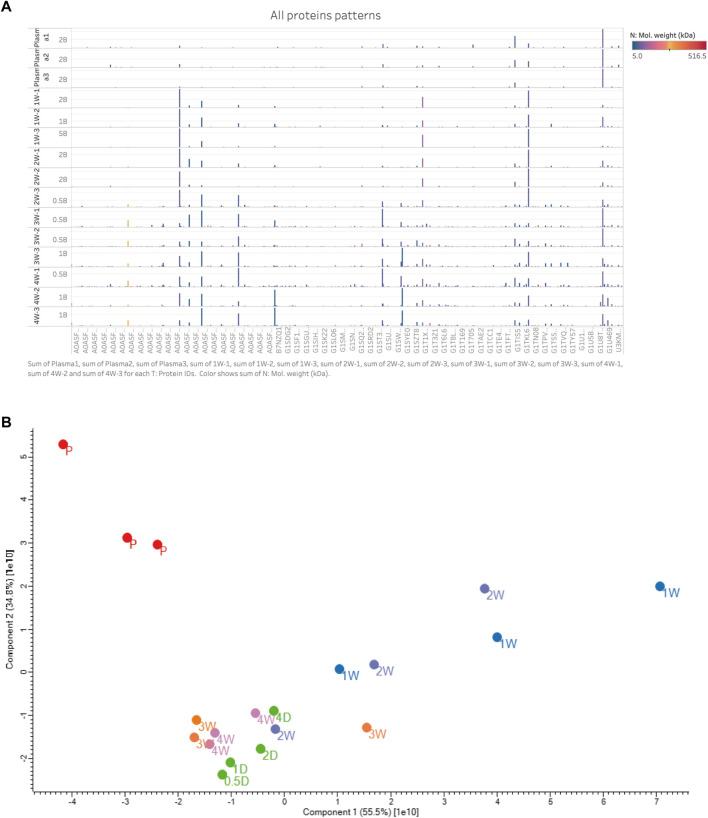
**(A)** All protein patterns with replicated samples. The *y*-axis was the 12 stable neointima samples (day 7, 14, 21, and 28, each *n* = 3) and 3 plasma samples (up 3 samples); The *x*-axis was partial the proteins’ gene name (too many to display); The protein profiles of neointima were not correlated with the plasma. And late-stage samples (3w and 4w) show more quantitation and types of proteins than others. Most abundance proteins in neointima were with low molecule weight. Color means the molecule weight. Hight is the proteins expressed quantitation. Protein’s name is not shown. **(B)** PCA map of all the samples in plasma and neointima groups.

After implantation, all 24 decellularized BIMA developed neointima on the luminal surface. The neointima was separated from the graft before proteomics analysis was performed. Based on the histological findings, we divided the specimens into three phases over the course of 28 days: I: acute phase (acute inflammatory thrombotic phase with unstable neointima, 0.5D-4D specimens); II: early-stage phase (acute inflammatory cells subsided and started to stabilize the intimal structure, 1 W specimens); and III: late-stage phase (ECM remodeling, 3–4 W specimens). In the neointima formed in just 4 weeks, over 1000 proteins were discovered. These proteins were detected with a broad molecular weight range from 5 to 516 kDa [Figure 4A]. Detailed data for all samples (Supplementary Material).

**FIGURE 5:**
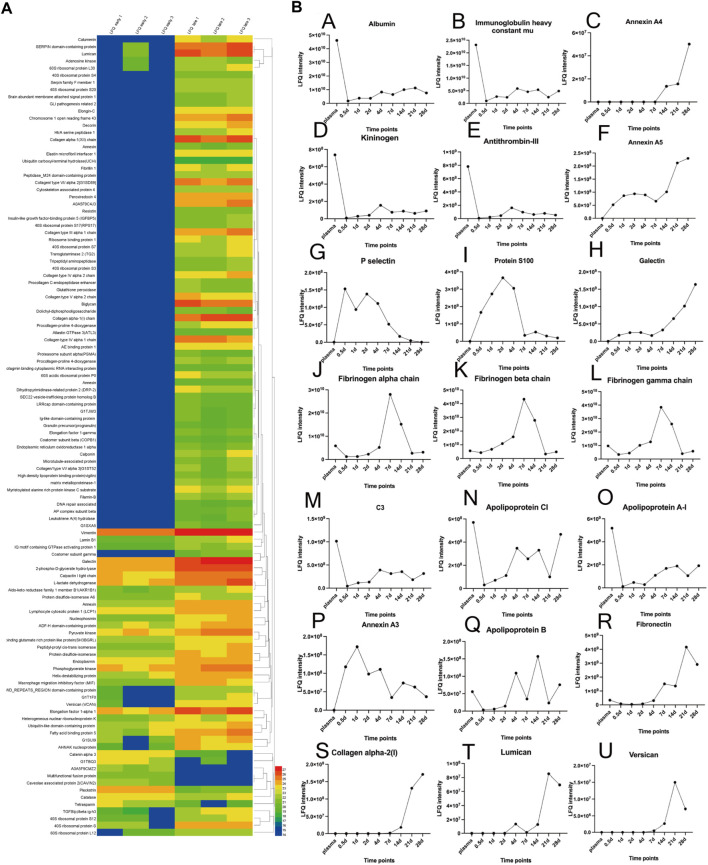
**(A)**: Protein profile of differential expressed proteins in late-stage neointima compared to the early. The heatmap showed hierarchical clustering results. The color bars represent the relative quantiles and the color range map indicates the protein expression ratios: blue boxes the down-regulated proteins, red ones the up-regulated proteins. **(B)**: Relative quantities of 21 proteins over time (including plasma); The (LFQ, label-free quantitation).

The principal component analysis (PCA) demonstrated a rather distinct distribution between plasma and neointima samples at various time points. All the neointimal proteomes were clearly different from plasma [Figure 4B]. The changes in the neointima after graft implantation were compared at eight different time points. Obviously, we discovered that the proportion of binding proteins altered over time [Figure 5]. We found some proteins with increased or decreased quantitation as a result of the Vroman effect throughout the course of 4 weeks [Figure 5II: A, B, C, F, H, O] (Vroman, 1987). Interestingly, several protein binding modes unexpectedly identified several proteins characterized by low abundance at the beginning of plasma exposure and later time points, but showing peak abundance at intermediate time points [Figure 5II: G, I, J, K, L, P]. During the acute phase, proteins that regulate hemostasis (e.g., fibrinogen, antithrombin-III, and plasminogen) [Figure 5II: J-L, E] and inflammation (e.g., Protein S100, P-selection, annexin) [Figure 5II: I, G, C, F] were increased, along with the discovery of lipoproteins on lipoprotein particles, including apolipoproteins A-I, C-I, and A-IV.

Overall, this represents a vigorous proteome response that emerges within a few hours after vascular implantation, driven in part by inflammatory cells (leukocytes) infiltrating with adhesive proteins (Fibronectin, P-selectin).

### In stable phase: Early versus late stage (II vs. III)

**FIGURE 6:**
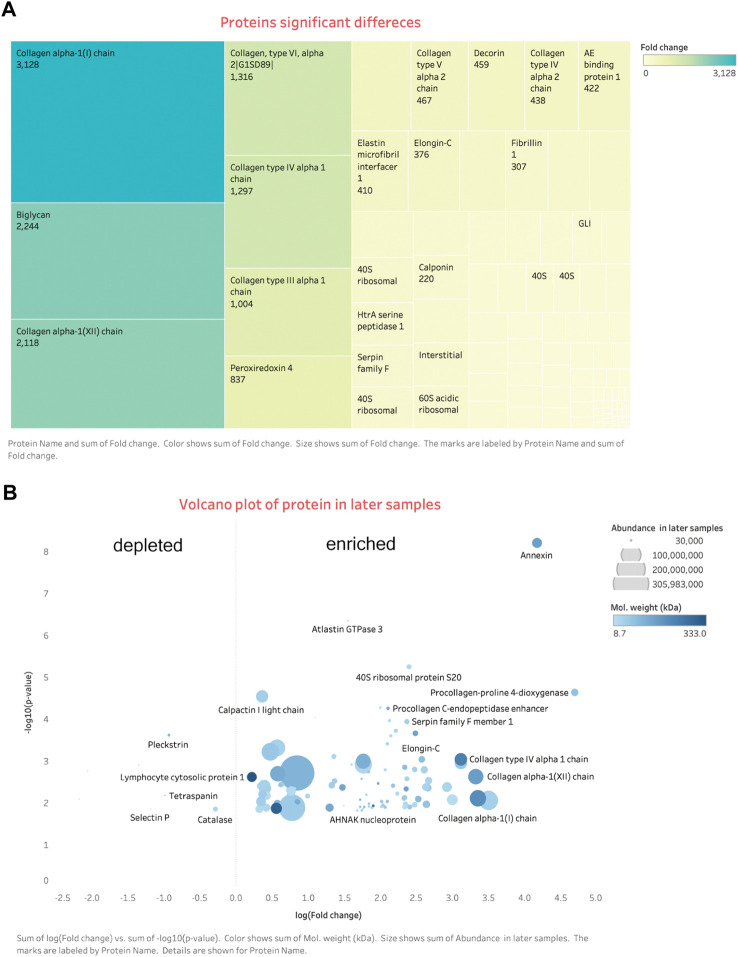
Differential expression proteins. **(A)** Tree map: shows most enriched proteins are the ECM related. **(B)** Volcano plot: on the right side are the enriched proteins, left are the depleted. More details signs are in the bottom of the figure.

And 110 proteins (7 proteins down-regulated and 103 up-regulated) were found to be differentially expressed in late-stage neointima (3–4 weeks samples) compared with the early samples (1-week neointima) (Details in Additional file: differential proteins). Hierarchical clustering analysis was conducted on the proteins, and the heatmap obtained from the analysis provided protein profiles across the late sample and early samples. [Figure 5, Figures 6A, B]. Proteins that respond in the late phase include fibrillar collagens (e.g., type I, III, and V), large aggregated proteins polysaccharides (e.g., versican, lumincan), small leucine-rich proteoglycan (e.g., biglycan) and intracellular proteins (e.g., vimentin). Proteins involved in the immunological response, cell adhesion, cell activation, extracellular matrix (ECM), proliferation, and migration are up-regulated by neointima development and subsequent remodeling [Figure 5I].

#### Gene ontology (GO) functional analysis

**FIGURE 7:**
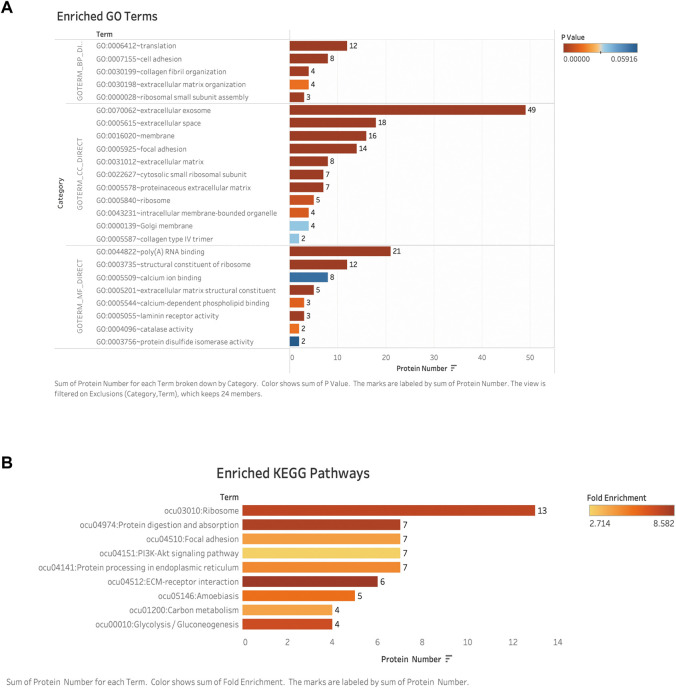
Bioinformatic analysis: **(A)** GO enriched analyses of differentially expressed proteins (DAPs) in late-stage neointima. The *y*-axis was the protein functional classification of GO: biological process (BP), cellular components (CC), and molecular function (MF). The *x*-axis was the number of proteins in each classification. Different colors indicated different P-valves. **(B)** Enriched pathways according to KEGG functional classification analyses of DAPs. The *y*-axis was the significantly enriched KEGG pathways. The *x*-axis was the number of DAPs contained in each KEGG pathway. Different colors indicated the different fold enrichment. DAPs: differentially expressed proteins; GO: Gene Ontology; KEGG: Kyoto Encyclopedia of Genes and Genomes.

GO analysis includes a biological process (BP), cellular component (CC), and molecular function (MF). To classify the differentially expressed proteins, we performed GO analysis of these proteins, and we defined categories with *p*-value <0.05 and fold enrichment> 2 as available results. Our results showed the majority of the differential expressed proteins were involved in translation (*n* = 12), cell adhesion (*n* = 8), and collagen fibril/extracellular matrix organization (*n* = 4) (as the main biological processes (*p* < 0.05)) [Figure 7A]. These results indicated that the predominant functions of differentially expressed proteins were mainly distributed in the extracellular space and were mainly correlated with the extracellular matrix regeneration or the processes of fibrosis. Detailed information on molecular functions, cellular components, and biological processes are displayed in additional files [Additional file. 4 GO terms].

### KEGG biological pathway analysis

Identified different expressed proteins from neointima were involved in a total of 9 KEGG biological pathways. Among these, the top six significantly enriched pathways were ribosome, protein digestion/absorption, focal adhesion, PI3K-Akt signaling pathway, protein processing in the endoplasmic reticulum, ECM-receptor interaction, carbon metabolism, and glycolysis/gluconeogenesis were among the most enriched pathways. [Figure 7B]. The ECM-receptor interaction and protein digestion/absorption were found to be significantly enriched. (Fold enrichment >8). Details of the KEGG pathway enrichment are shown in the additional file [Additional file KEGG].

#### Protein-proteins interaction analysis

**FIGURE 8:**
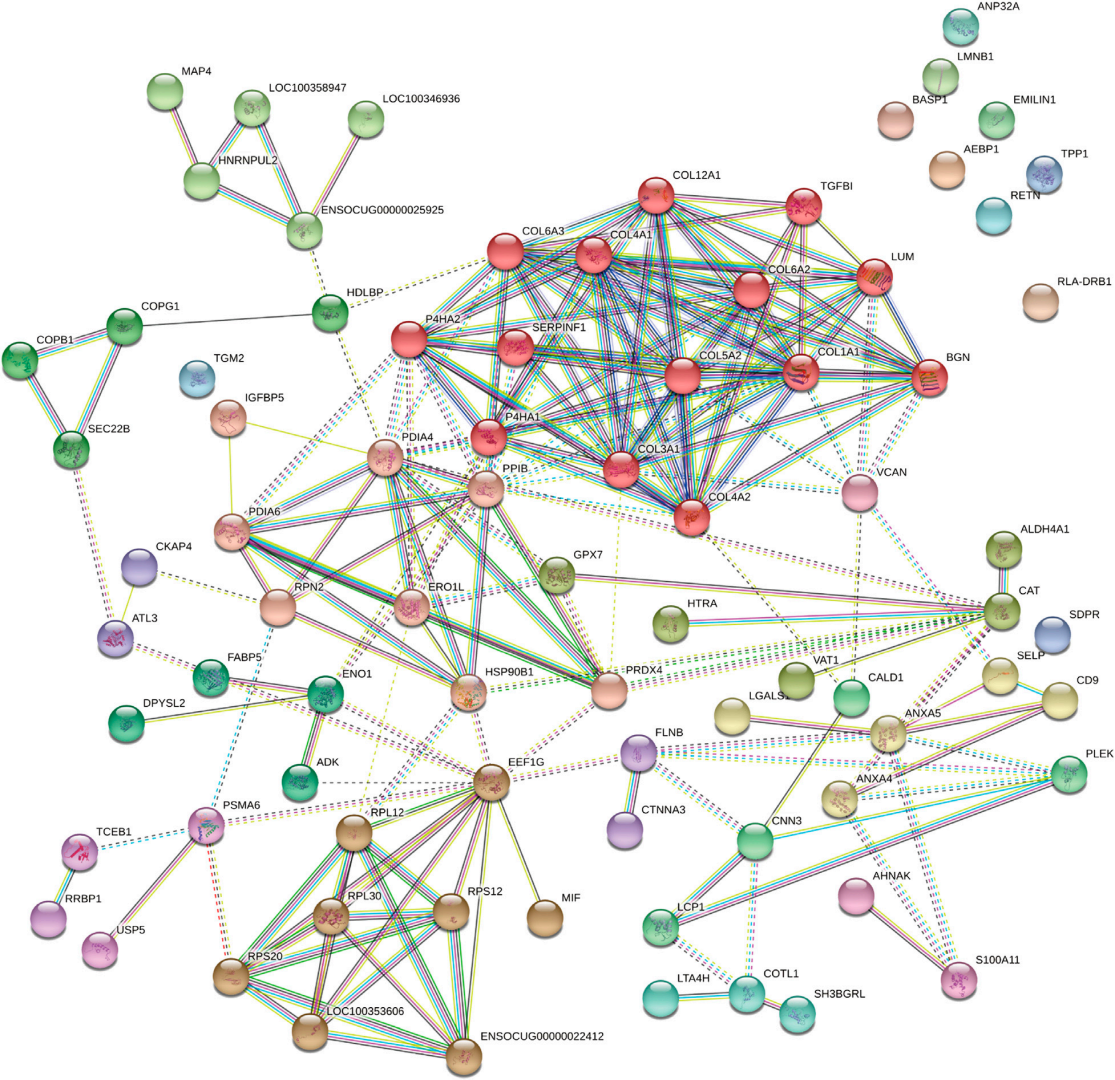
The protein-protein interaction network of DAPs analyzed by the STRING software. Each note represents a protein and lines represent interactions. DAPs: differentially expressed proteins.

The protein-protein interaction network was analyzed using the publicly available program STRING and the results are shown in [Figure 8]. Based on interaction analysis, these 76 proteins are predicted to have direct interactions with 110 proteins that are differentially expressed. Interestingly, the main proteins (e.g., Collagen helix, versican, and biglycan) were involved in multiple pathways, such as protein digestion/absorption, focal adhesion, PI3K-Akt signaling pathway, and protein processing in the endoplasmic reticulum and ECM-receptor interaction [Details in additional file bioinformatic]. Therefore, neointima showed the regenerative processes in the late stage.

## Discussion

Previous studies of explanted vascular grafts have demonstrated the host cell response which determines the fate of the implanted vascular graft (Kirkton et al., 2019). Most research focused on re-endothelialization, mimicking native vessels and regeneration ability (Yuan et al., 2020) (Shi et al., 2019) (Shafiq et al., 2018). Here, this research has various significant characteristics. To begin, we employed a sequential neointima observation in conjunction with proteomics, departing from the traditional focus on regenerative grafts. We uncover previously unknown neointima components and give valuable insights into neointima development via three stages of remodeling using this advanced method. In a decellularized TEVGs replacement model, the extensive proteomic comparison enabled for the first time a combined investigation of proteins and cells with the involvement of related biological processes during the early and late stages of neointima development.

### Roles of protein adsorption in plasma

Protein adsorption is the very first step in the process of the biomaterial occurring within seconds (Baier and Dutton, 1969). The process of protein adsorption is determined by the biomaterial, its hydrophobicity, surface charge, topology, and bio-chemically reactive sites (Xu et al., 2014). Once initiated, the biological cascade process is continuous, especially the thrombosis formation, platelet activation, and acute information (Brash et al., 2019) (Jaffer and Weitz, 2019) (Gorbet et al., 2019) (Gorbet et al., 2019), even after the endothelial cells layer has been formed, leading to intimal hyperplasia with the cell’s infiltration, pathological proliferation and extracellular matrix deposition (Watase et al., 1992). Because of the inflammation and accumulations of the extracellular matrix, the normal processes of vascularization and angiogenesis may be disrupted.

Indeed, it has been demonstrated that protein layers are formed typically within a few seconds (Baier and Dutton, 1969). Based on our studies, it should take hours for the formation of a whole protein layer in the vascular lumen [Figure 2A]. There is evidence that the composition of the protein layer formed at the plasma interface varies with time (Tenzer et al., 2013). Vroman proposed that in plasma, protein adsorption is sequential, with enriched proteins with lower surface activity being replaced by proteins with lower surface activity over time (Vroman, 1987). As predicted by the Vorman effect, the proteome we observed showed an increase or decrease in binding over time (During the acute phase or partial late stage), implying that these proteins were being replaced by other proteins (P-selectin and Protein S100) [Figure 5II: G, I]. However, our results showed that most proteins composition enriched in the neointima at different times was not correlated with the plasma protein abundance [Figures 4A, 5II]; Furthermore, the proteins binding modes unexpectedly identified several proteins characterized by low abundance at the beginning of plasma exposure and later time points, but showing peak abundance at intermediate time points (Fibrinogen) [Figure 5II: J-L]. In all, the protein layer adsorbed from blood is complex with multiple forces.

The early proteins play an important role in providing sites of cell adhesion and influence cell behavior and signaling. Our proteomics data comparison of the three-stage neointima tissue revealed the following findings: First, among all the proteins data sets, we found that the amounts of adsorbed proteins changed significantly over time. Novel binding kinetics for biological relevant proteins, which cannot be explained solely by the Vroman effect (Vroman, 1987), the cells are involved (inflammatory cells, fibroblast, and SMC-like cells); Second, protein binding did not simply correlate with their relative abundance in the plasma. Albumin and fibrinogen were the most abundant proteins detected in all the neointima samples; Third, neither protein size nor charge significantly determined the protein fingerprints, electrostatic effects alone do not constitute the major driving force. Immune response and thrombi formation relative proteins were abundant in the neointima tissue all the time, though the quantitative amount change slightly. This may also explain the unformed functional ECs layer on the in neointima until day 7.

### Changes in the neointima

Our proteomic data comparison allowed an analysis of neointima components during different stages of tissue remodeling in response to the implanted graft. The acute phase (in the first week) response to the biomaterial was related to the type of the material. Based on our decellularized graft, two different functional classes were increased: 1). Proteins involved in platelet activation and thromboses, such as fibrinogen, kininogen, antithrombin-III, and plasminogen; 2). Inflammatory-related proteins, such as annexin A1/4/5/11, protein S100, and P-selectin, implicate the recruitment of inflammatory cells (leukocyte, monocyte, and macrophage) to the site of implantation. P-selectin, for example, plays a key role in mediating inflammation by promoting adherence of leukocytes to activated platelets. Hayashi’s study showed inhibition of P-selectin-mediated leukocyte recruitment may prevent the development of neointima formation on a balloon-injured model (Hayashi et al., 2000). This may also explain why P-selection appearances high abundance in neointima until day 7 [Figure 5II: G]. It is correlated with the leukocyte migration in the first week [Figure 5II: G]. Various apolipoproteins, such as Apolipoprotein A-I, and Apolipoprotein A-IV, are present in atherosclerosis formation. We observed proteins that were involved in platelet activation, coagulation, and inflammation kept high abundance in neointima. These proteins are the evidence for the thrombosis, inflammation, and biological adsorption processes activated all the time. The neointima was remodeling in our observation, its stage had been stable after day 7 when the leukocytes faded. Furthermore, the time was the most thickness of the neointima. Stenosis was first recognized in 1 week [Figure 2] and has largely been attributed to protein adsorption, leukocyte recruitment, and activation of the coagulation cascade. Therefore, shorting the accumulation of leukocytes (e.g., inhibiting the p-selectin) may be the potential target to impair or disrupt the acute form of the neointima. It should be clarified if these proteins might be involved in the accelerated course of neointima formation within the vascular graft.

### Neointima remodeling over time with ECMs regeneration

In the stable phase of neointima (1–4 weeks), ECM protein changes are predominantly after 2 weeks combined with our histological findings, and it is the time myofibroblasts/fibroblasts or SMC-like cells synthesize new ECM proteins. (Figure 6A). A significant increase after 3 weeks was seen in fibrillar collagens, biglycan, and lumican. These matrix proteins and involved in cellular functions such as collagen fibril assembly, cell migration, proliferation, and fibrosis. In general, neointima shows regeneration with cells and ECMs in the late stage. We can simplify the fate of implanted vascular graft: the process of the formation of the neointima tissue transformation. Step 1: early protein adsorption, leukocytes binding, and thrombi formation; Step 2: Increasing the thickness (more proteins and leukocytes accumulation) and cells polarization; Step 3: Autolysis of leukocytes and appearance of macrophages; Step 4: Fibroblasts/myofibroblasts and SMC-like cells popping up and decreasing of the protein adsorption; Step 5: Collagen fibrils production and cells perforation. And finally, neointima acts as the base matrix after 4-weeks of remodeling in our study [Figures 2, 3]. It was seen the same neointima-transformed matrix layer in the ePTFE graft after being implanted in a patient for 7 years [Additional file 6: Supplementary Figure S4]. Neointima has an impact on the lumen area and the surface modification, but it shows the potential for regeneration.

### Targets for regulation of neointima

For decades, investigation of tissue-engineered vascular graft (TEVG) has persisted on anti-thrombin strategies and promoting *in situ* regeneration (ECs and SMCs) with significant advancement in short patency. (Yuan et al., 2020). In the long term, the grafts showed the low-rate patency duo to the intima hyperplasia. So, we should take more effort to overcome the challenges faced in TEVG development. In summary, neointima formation occurs in TEVG implantation because of multiple biological activities including protein adsorption, leukocyte recruitment, activation of the coagulation cascade (hours to days), macrophages/fibroblasts/SMC-like cells migration, and extracellular matrix deposition causing stenosis. The whole pathological patterns are similar to the neointima development in coronary artery bypass graft and vascular injury models. The significant characteristics of the TEVGs are the absence of functional ECs on the lumen and SMCs in the media. Consequently, TEVGs implantation causes protein adsorption, thrombi formation, and later events leading to the failure of TEVGs without ECs layers.

Our study highlights the effects of serum proteins adsorption and host cells responding to the biomaterial and the neointima tissue remodeling after graft implantation. The serum proteins adsorption on the vascular graft and its correlation with subsequent bio-activities have not been studied. And this layer critically affects the luminal diameter of the small-diameter vascular graft. Previous research has shown evidence of the proteins (Ubiquitin carboxyl-terminal hydrolase, WD_REPEATS_REGION domain-containing protein, TGFBIp/beta ig-h3) with the effects to promote the neointima formation or hyperplasia process in autologous graft. (Takami et al., 2007) (Hu et al., 2020) (Kim et al., 2009).

Most of the potentially related proteins are listed in Additional file 6, such as fibrinogen, P-selectin, annexin, resistin, protein S100, and galectin (all involved in affecting the inflammation, thrombosis, and SMCs activities). These may be also the targets to regulate the neointima in the TEVG model aiming at these aspects: 1. Reducing protein adsorption and anti-platelet; 2. Acute inflammation (reducing the neutrophil accumulation); 3. Inhibition of excessive collagen deposit. The molecular and cellular mechanisms may relate to inflammation (leukocyte), thrombosis contributes to the early formation of neointima, and macrophages, fibroblasts/SMCs may influence the process of neointima hyperplasia. (Gan et al., 2013). The specific underlined mechanisms are remained to be studied further. Furthermore, the TEVG graft is lack of SMCs in the media. Where did the SMC-like come from?.

### Pros and cons

A strength of our study is the advantages of the proteomics technique. In a decellularized TEVGs replacement model, the extensive proteomic comparison enabled for the first time a combined investigation of proteins and cells with the involvement of related biological processes during the early and late stages of neointima development. In the rabbit abdominal artery replacement model, it is the first time found that serum proteins adsorbed and acute inflammatory cell migration are considered essential for neointima formation after graft implantation.

However, one disadvantage of dealing with rabbit tissue is the insufficiency of antibodies, therefore proteomics was essential for a complete protein analysis free of antibody restrictions. The endothelization was not observed in all neointima samples. We should explore the graft's longer implantation time point (at least 6 months) to understand the neointima's further remodeling.

## Conclusion

Using a rabbit model of acellular small-caliber TEVG implantation and state-of-the-art mass spectrometry, we provide the first proteomic characterization of neointima formation in 4 weeks. Our study revealed the differences in early- and late-stage stabled neointima of different types and relative amounts of proteins. The significantly up-regulation proteins functioned in several biological processes: digestion/absorption, focal adhesion, PI3K-Akt signaling pathway, protein processing in the endoplasmic reticulum, ECM-receptor interaction, carbon metabolism, and glycolysis/gluconeogenesis. And in the acute unstable phase, the loose and uniform neointima was mainly composed of plasma proteins and inflammatory cells. This discovery provides new insights for us to know the molecular and cellular mechanisms that contribute to the formation of neointima that may cause graft stenosis. At the very least, the neointima should be considered as a two-sided sword, which may lead to graft early failure or the base of the regenerative media. On this basis, we will further extend the animal experiments in future studies to demonstrate regenerable endothelial cells on the neointima and explore the construction of new TEVGs to promote neointima non-pathological remodeling (through reducing protein adsorption, regulation of acute inflammation, inhibition of late fibrosis) on long-term patency of artificial vessels.

## Data Availability

The original contributions presented in the study are included in the article/Supplementary Material, further inquiries can be directed to the corresponding author

## References

[B1] Anderson J. M. Rodriguez A. Chang D. T. (2008). Foreign Body Reaction to Biomaterials. Semin. Immunol. 20 (2), 86–100. 10.1016/j.smim.2007.11.004 18162407 PMC2327202

[B2] Baier R. E. Dutton R. C. (1969). Initial Events in Interactions of Blood with a Foreign Surface. J. Biomed. Mat. Res. 3 (1), 191–206. 10.1002/jbm.820030115 5784964

[B3] Brash J. L. Horbett T. A. Latour R. A. Tengvall P. (2019). The Blood Compatibility Challenge. Part 2: Protein Adsorption Phenomena Governing Blood Reactivity. Acta Biomater. 94, 11–24. 10.1016/j.actbio.2019.06.022 31226477 PMC6642842

[B4] Collins M. J. Li X. Lv W. Yang C. Protack C. D. Muto A. (2012). Therapeutic Strategies to Combat Neointimal Hyperplasia in Vascular Grafts. Expert Rev. cardiovasc. Ther. 10 (5), 635–647. 10.1586/erc.12.33 22651839 PMC3401520

[B5] Cox J. Hein M. Y. Luber C. A. Paron I. Nagaraj N. Mann M. (2014). Accurate Proteome-wide Label-free Quantification by Delayed Normalization and Maximal Peptide Ratio Extraction, Termed MaxLFQ. Mol. Cell. Proteomics 13 (9), 2513–2526. 10.1074/mcp.M113.031591 24942700 PMC4159666

[B6] Cox J. Mann M. (2008). MaxQuant Enables High Peptide Identification Rates, Individualized p.p.b.-range Mass Accuracies, and Proteome-wide Protein Quantification. Nat. Biotechnol. 26 (12), 1367–1372. 10.1038/nbt.1511 19029910

[B7] Davies M. G. Hagen P. O. (1995). Pathophysiology of Vein Graft Failure: a Review. Eur. J. Vasc. Endovasc. Surg. 9 (1), 7–18. 10.1016/s1078-5884(05)80218-7 7664016

[B8] Delius J. Trautmann S. Medard G. Kuster B. Hannig M. Hofmann T. (2017). Label-free Quantitative Proteome Analysis of the Surface-Bound Salivary Pellicle. Colloids Surfaces B Biointerfaces 152, 68–76. 10.1016/j.colsurfb.2017.01.005 28086104

[B9] Faries P. L. Logerfo F. W. Arora S. Pulling M. C. Rohan D. I. Akbari C. M. (2000). Arm Vein Conduit Is Superior to Composite Prosthetic-Autogenous Grafts in Lower Extremity Revascularization. J. Vasc. Surg. 31 (6), 1119–1127. 10.1067/mva.2000.106488 10842148

[B10] Gan A. M. Pirvulescu M. M. Stan D. Simion V. Calin M. Manduteanu I. (2013). Monocytes and Smooth Muscle Cells Cross-Talk Activates STAT3 and Induces Resistin and Reactive Oxygen Species and Production. J. Cell. Biochem. 114 (10), 2273–2283. 10.1002/jcb.24571 23606279

[B11] Glickman M. E. Rao S. R. Schultz M. R. (2014). False Discovery Rate Control Is a Recommended Alternative to Bonferroni-type Adjustments in Health Studies. J. Clin. Epidemiol. 67 (8), 850–857. 10.1016/j.jclinepi.2014.03.012 24831050

[B12] Gong W. Lei D. Li S. Huang P. Qi Q. Sun Y. (2016). Hybrid Small-Diameter Vascular Grafts: Anti-expansion Effect of Electrospun Poly Epsilon-Caprolactone on Heparin-Coated Decellularized Matrices. Biomaterials 76, 359–370. 10.1016/j.biomaterials.2015.10.066 26561933

[B13] Gorbet M. Sperling C. Maitz M. F. Siedlecki C. A. Werner C. Sefton M. V. (2019). The Blood Compatibility Challenge. Part 3: Material Associated Activation of Blood Cascades and Cells. Acta Biomater. 94, 25–32. 10.1016/j.actbio.2019.06.020 31226478

[B14] Hayashi S. Watanabe N. Nakazawa K. Suzuki J. Tsushima K. Tamatani T. (2000). Roles of P-Selectin in Inflammation, Neointimal Formation, and Vascular Remodeling in Balloon-Injured Rat Carotid Arteries. Circulation 102 (14), 1710–1717. 10.1161/01.cir.102.14.1710 11015352

[B15] Herrmann F. E. M. Lamm P. Wellmann P. Milz S. Hagl C. Juchem G. (2019). Autologous Endothelialized Vein Allografts in Coronary Artery Bypass Surgery - Long Term Results. Biomaterials 212, 87–97. 10.1016/j.biomaterials.2019.05.019 31108275

[B16] Hu J. Pi S. Xiong M. Liu Z. Huang X. An R. (2020). WD Repeat Domain 1 Deficiency Inhibits Neointima Formation in Mice Carotid Artery by Modulation of Smooth Muscle Cell Migration and Proliferation. Mol. Cells 43 (8), 749–762. 10.14348/molcells.2020.0085 32868491 PMC7468582

[B17] Jaffer I. H. Weitz J. I. (2019). The Blood Compatibility Challenge. Part 1: Blood-Contacting Medical Devices: The Scope of the Problem. Acta Biomater. 94, 2–10. 10.1016/j.actbio.2019.06.021 31226480

[B18] Kim H. J. Kim P. K. Bae S. M. Son H. N. Thoudam D. S. Kim J. E. (2009). Transforming Growth Factor-β–Induced Protein (TGFBIp/β Ig-H3) Activates Platelets and Promotes Thrombogenesis. Blood 114 (25), 5206–5215. 10.1182/blood-2009-03-212415 19738031

[B19] Kirkton R. D. Santiago-Maysonet M. Lawson J. H. Tente W. E. Dahl S. L. M. Niklason L. E. (2019). Bioengineered Human Acellular Vessels Recellularize and Evolve into Living Blood Vessels after Human Implantation. Sci. Transl. Med. 11 (485), eaau6934. 10.1126/scitranslmed.aau6934 30918113 PMC7557107

[B20] Lacolley P. Regnault V. Nicoletti A. Li Z. Michel J. B. (2012). The Vascular Smooth Muscle Cell in Arterial Pathology: a Cell that Can Take on Multiple Roles. Cardiovasc. Res. 95 (2), 194–204. 10.1093/cvr/cvs135 22467316

[B21] Li Z. K. Wu Z. S. Lu T. Yuan H. Y. Tang H. Tang Z. J. (2016). Materials and Surface Modification for Tissue Engineered Vascular Scaffolds. J. Biomaterials Sci. Polym. Ed. 27 (15), 1534–1552. 10.1080/09205063.2016.1217607 27484610

[B22] Liu Y. Chen C. Xie X. Yuan H. Tang Z. Qian T. (2022). Photooxidation and Pentagalloyl Glucose Cross-Linking Improves the Performance of Decellularized Small-Diameter Vascular Xenograft *In Vivo* . Front. Bioeng. Biotechnol. 10, 816513. 10.3389/fbioe.2022.816513 35402413 PMC8987116

[B23] Lu W. D. Zhang M. Wu Z. S. Hu T. H. (2009). Decellularized and Photooxidatively Crosslinked Bovine Jugular Veins as Potential Tissue Engineering Scaffolds. Interact. Cardiovasc. Thorac. Surg. 8 (3), 301–305. 10.1510/icvts.2008.194076 19074454

[B24] Lu W. D. Zhang M. Wu Z. S. Hu T. H. Xu Z. J. Liu W. (2010). The Performance of Photooxidatively Crosslinked Acellular Bovine Jugular Vein Conduits in the Reconstruction of Connections between Pulmonary Arteries and Right Ventricles. Biomaterials 31 (10), 2934–2943. 10.1016/j.biomaterials.2009.12.046 20053442

[B25] Ma L. Y. Chen W. W. Gao R. L. Liu L. S. Zhu M. L. Wang Y. J. (2020). China Cardiovascular Diseases Report 2018: an Updated Summary. J. Geriatr. Cardiol. 17 (1), 1–8. 10.11909/j.issn.1671-5411.2020.01.001 32133031 PMC7008101

[B26] Mozaffarian D. Benjamin E. J. Go A. S. Arnett D. K. Blaha M. J. Cushman M. (2016). Heart Disease and Stroke Statistics-2016 Update: A Report from the American Heart Association. Circulation 133 (4), e38–360. 10.1161/CIR.0000000000000350 26673558

[B27] Muto A. Model L. Ziegler K. Eghbalieh S. D. Dardik A. (2010). Mechanisms of Vein Graft Adaptation to the Arterial Circulation: Insights into the Neointimal Algorithm and Management Strategies. Circ. J. 74 (8), 1501–1512. 10.1253/circj.cj-10-0495 20606326 PMC3662001

[B28] Reinhardt J. W. Rosado J. D. R. Barker J. C. Lee Y. U. Best C. A. Yi T. (2019). Early Natural History of Neotissue Formation in Tissue-Engineered Vascular Grafts in a Murine Model. Regen. Med. 14 (5), 389–408. 10.2217/rme-2018-0133 31180275 PMC6587105

[B29] Riascos-Bernal D. F. Chinnasamy P. Gross J. N. Almonte V. Egana-Gorrono L. Parikh D. (2017). Inhibition of Smooth Muscle Beta-Catenin Hinders Neointima Formation after Vascular Injury. Arterioscler. Thromb. Vasc. Biol. 37 (5), 879–888. 10.1161/ATVBAHA.116.308643 28302627 PMC5408313

[B30] Ross R. Glomset J. A. (1973). Atherosclerosis and the Arterial Smooth Muscle Cell: Proliferation of Smooth Muscle Is a Key Event in the Genesis of the Lesions of Atherosclerosis. Science 180 (4093), 1332–1339. 10.1126/science.180.4093.1332 4350926

[B31] Roy-Chaudhury P. Wang Y. Krishnamoorthy M. Zhang J. Banerjee R. Munda R. (2009). Cellular Phenotypes in Human Stenotic Lesions from Haemodialysis Vascular Access. Nephrol. Dial. Transpl. 24 (9), 2786–2791. 10.1093/ndt/gfn708 PMC2734173 19377054

[B32] Shafiq M. Zhang Q. Zhi D. Wang K. Kong D. Kim D. H. (2018). *In Situ* Blood Vessel Regeneration Using SP (Substance P) and SDF (Stromal Cell–Derived Factor)-1α Peptide Eluting Vascular Grafts. Arterioscler. Thromb. Vasc. Biol. 38 (7), e117–e134. 10.1161/ATVBAHA.118.310934 29853570 PMC6039427

[B33] Shi J. Chen S. Wang L. Zhang X. Gao J. Jiang L. (2019). Rapid Endothelialization and Controlled Smooth Muscle Regeneration by Electrospun Heparin-Loaded Polycaprolactone/gelatin Hybrid Vascular Grafts. J. Biomed. Mat. Res. 107 (6), 2040–2049. 10.1002/jbm.b.34295 30556953

[B34] Suna G. Wojakowski W. Lynch M. Barallobre-Barreiro J. Yin X. Mayr U. (2018). Extracellular Matrix Proteomics Reveals Interplay of Aggrecan and Aggrecanases in Vascular Remodeling of Stented Coronary Arteries. Circulation 137 (2), 166–183. 10.1161/CIRCULATIONAHA.116.023381 29030347 PMC5757669

[B35] Szklarczyk D. Franceschini A. Wyder S. Forslund K. Heller D. Huerta-Cepas J. (2015). STRING V10: Protein-Protein Interaction Networks, Integrated over the Tree of Life. Nucleic Acids Res. 43, D447–D452. 10.1093/nar/gku1003 25352553 PMC4383874

[B36] Takami Y. Nakagami H. Morishita R. Katsuya T. Cui T. X. Ichikawa T. (2007). Ubiquitin Carboxyl-Terminal Hydrolase L1, a Novel Deubiquitinating Enzyme in the Vasculature, Attenuates NF-Κb Activation. Arterioscler. Thromb. Vasc. Biol. 27 (10), 2184–2190. 10.1161/ATVBAHA.107.142505 17690318

[B37] Tenzer S. Docter D. Kuharev J. Musyanovych A. Fetz V. Hecht R. (2013). Rapid Formation of Plasma Protein Corona Critically Affects Nanoparticle Pathophysiology. Nat. Nanotechnol. 8 (10), 772–781. 10.1038/nnano.2013.181 24056901

[B38] Tsao C. W. Aday A. W. Almarzooq Z. I. Alonso A. Beaton A. Z. Bittencourt M. S. (2022). Heart Disease and Stroke Statistics-2022 Update: A Report from the American Heart Association. Circulation 145 (8), e153–e639. 10.1161/CIR.0000000000001052 35078371

[B39] Tyanova S. Temu T. Sinitcyn P. Carlson A. Hein M. Y. Geiger T. (2016). The Perseus Computational Platform for Comprehensive Analysis of (Prote)omics Data. Nat. Methods 13 (9), 731–740. 10.1038/nmeth.3901 27348712

[B40] Virani S. S. Alonso A. Benjamin E. J. Bittencourt M. S. Callaway C. W. Carson A. P. (2020). Heart Disease and Stroke Statistics-2020 Update: A Report from the American Heart Association. Circulation 141 (9), e139–e596. 10.1161/CIR.0000000000000757 31992061

[B41] Vroman L. (1987). The Importance of Surfaces in Contact Phase Reactions. Semin. Thromb. Hemost. 13 (1), 79–85. 10.1055/s-2007-1003477 3551077

[B42] Watase M. Kambayashi J. Itoh T. Tsuji Y. Kawasaki T. Shiba E. (1992). Ultrastructural Analysis of Pseudo-intimal Hyperplasia of Polytetrafluoroethylene Prostheses Implanted into the Venous and Arterial Systems. Eur. J. Vasc. Surg. 6 (4), 371–380. 10.1016/s0950-821x(05)80282-8 1499739

[B43] Westerband A. Gentile A. T. Hunter G. C. Gooden M. A. Aguirre M. L. Berman S. S. (2000). Intimal Growth and Neovascularization in Human Stenotic Vein Grafts1. J. Am. Coll. Surg. 191 (3), 264–271. 10.1016/s1072-7515(00)00320-3 10989901

[B44] Wierer M. Werner J. Wobst J. Kastrati A. Cepele G. Aherrahrou R. (2021). A Proteomic Atlas of the Neointima Identifies Novel Druggable Targets for Preventive Therapy. Eur. Heart J. 42 (18), 1773–1785. 10.1093/eurheartj/ehab140 33829256 PMC8104955

[B45] Wynn T. A. Vannella K. M. (2016). Macrophages in Tissue Repair, Regeneration, and Fibrosis. Immunity 44 (3), 450–462. 10.1016/j.immuni.2016.02.015 26982353 PMC4794754

[B46] Xu L. C. Bauer J. W. Siedlecki C. A. (2014). Proteins, Platelets, and Blood Coagulation at Biomaterial Interfaces. Colloids Surfaces B Biointerfaces 124, 49–68. 10.1016/j.colsurfb.2014.09.040 25448722 PMC5001692

[B47] Yuan H. Chen C. Liu Y. Lu T. Wu Z. (2020). Strategies in Cell-free Tissue-Engineered Vascular Grafts. J. Biomed. Mat. Res. A 108 (3), 426–445. 10.1002/jbm.a.36825 31657523

[B48] Zhang S. M. Jiang L. Zhao X. Liu J. F. Liang B. Liu C. (2019). A Disintegrin and Metalloprotease 22 Accelerates Neointima Formation by Activating ERK Signaling. Atherosclerosis 283, 92–99. 10.1016/j.atherosclerosis.2019.02.002 30822685

