# Supplementary figures and images for: A proteomics analysis of neointima formation on decellularized vascular grafts reveals regenerative alterations in protein signature running head: Proteomics analysis of neointima formation
Source: Front Bioeng Biotechnol. 2022 Aug 30;10:894956. doi: 10.3389/fbioe.2022.894956 (PMC9673820; doi:10.3389/fbioe.2022.894956)

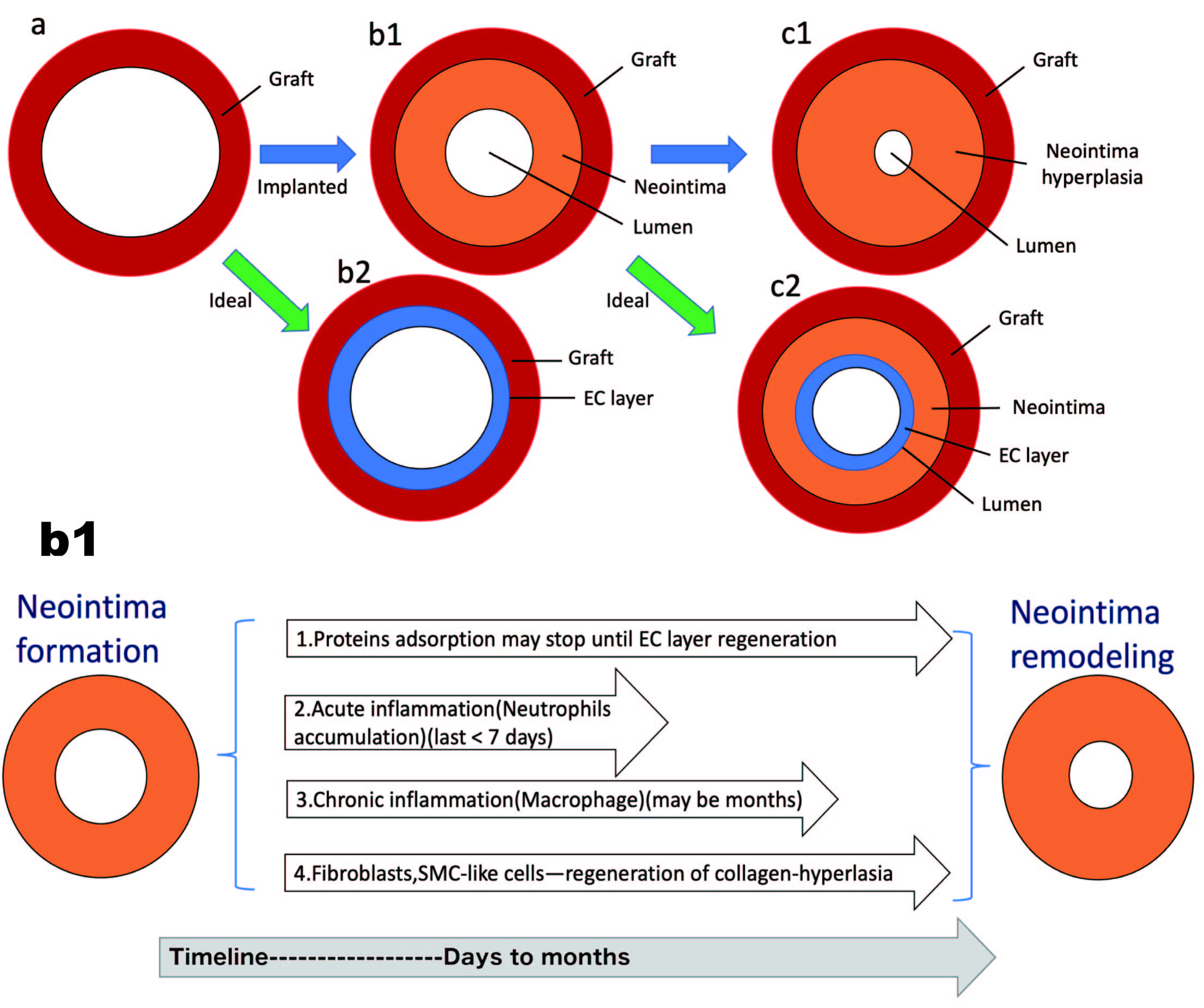

Supplement: Supplementary file 3 [file Image1.JPEG]
