# Supplementary material for: A proteomics analysis of neointima formation on decellularized vascular grafts reveals regenerative alterations in protein signature running head: Proteomics analysis of neointima formation
Source: Front Bioeng Biotechnol. 2022 Aug 30;10:894956. doi: 10.3389/fbioe.2022.894956 (PMC9673820; doi:10.3389/fbioe.2022.894956)
Supplement: Supplementary file 7 [file DataSheet1.docx]

**SUPPLEMENTAL MATERIAL**

1. **MaxQuant Detailed parameters settings:**

Details rules are as follows: Carbamidomethylated cysteine was selected as fixed modification and oxidation of methionine as well as N-terminal protein acetylation as variable modification. Trypsin/P was specified as the proteolytic enzyme with up to two missed cleavage sites allowed. Precursor tolerance was set to 6 ppm and fragment ion tolerance to 20 ppm. Peptide identifications required a minimal length of seven amino acids, and all data sets were adjusted to 1% protein FDR and 1% PSM.

**Figure 1B---supplemental details of the graft:**


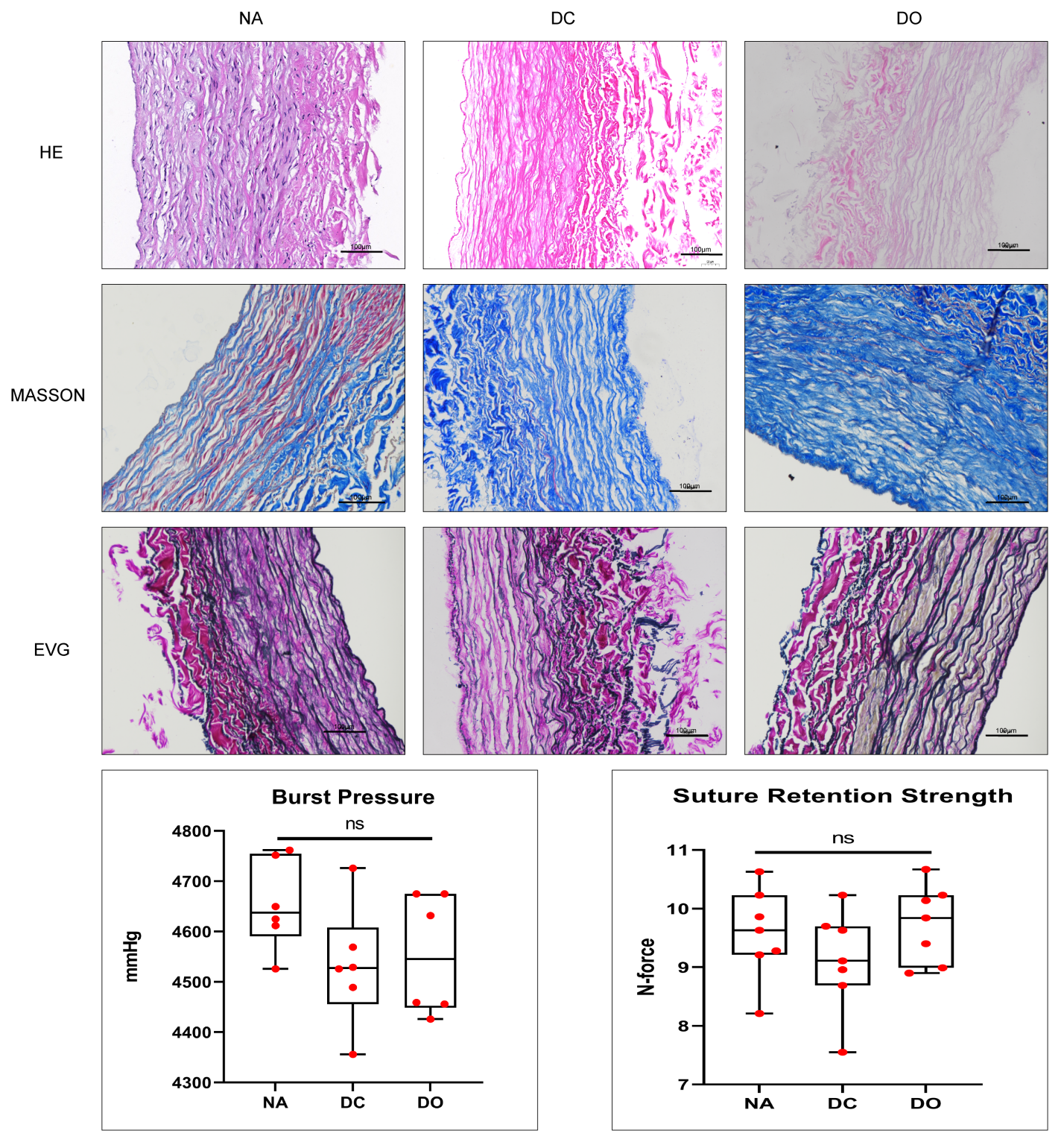
Figure S1: According to our previous study. The decellularized BIMA with a length of 10 to 15 cm and a diameter of 3 to 6 mm were obtained. The decellularized BIMA showed a complete internal elastin lamina membrane, thin media, which contained collagen and elastic fibers, and a relatively loose adventitia. The acellular BIMA has optimal biomechanical properties. Its burst pressure was as high as 4700 mmHg, and the suture retention reached approximately 9.0 N. which fully met the needs of human arterial blood pressure, and had good durability.

1. **Supplemental Figure S2 IHC with Negative control**


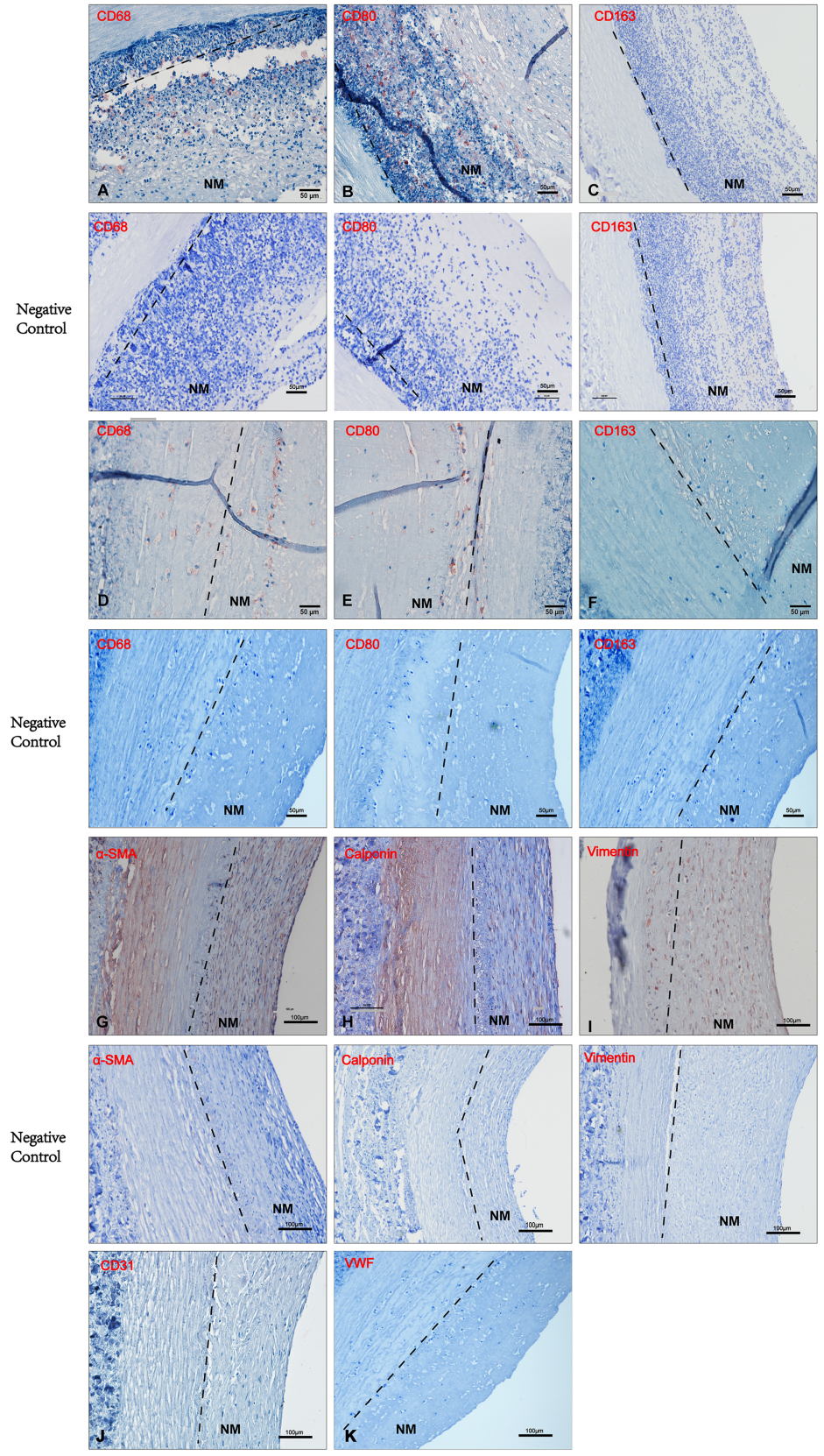


Figure S2: Immunohistochemical studies of the decellularized BIMA grafts explanted after implantation in vivo. Panels A, B, and C show staining for CD68 (+), CD80 (+), and CD163 (-) before day 7 samples (Below is the negative controls); Panels D, E, F, G, H, I, J, and K show the stable neointima samples during day 7 to 28 (Each panel below is the negative control). Three types of cells are seen CD68 (+) / CD80 (+) macrophages, αSMA (+)/calponin (+) SMC-like cells and vimentin (+) fibroblasts. No positive markers (CD31 and vWF) for ECs are seen. (small bar, 50μm; large bar, 100μm). vWF: von Willebrand Factor; αSMA: Smooth muscle alpha-actin; SMC: smooth muscle cell.

1. **Supplemental figure S3 MPO+ and LY6G+ neutrophils**

**
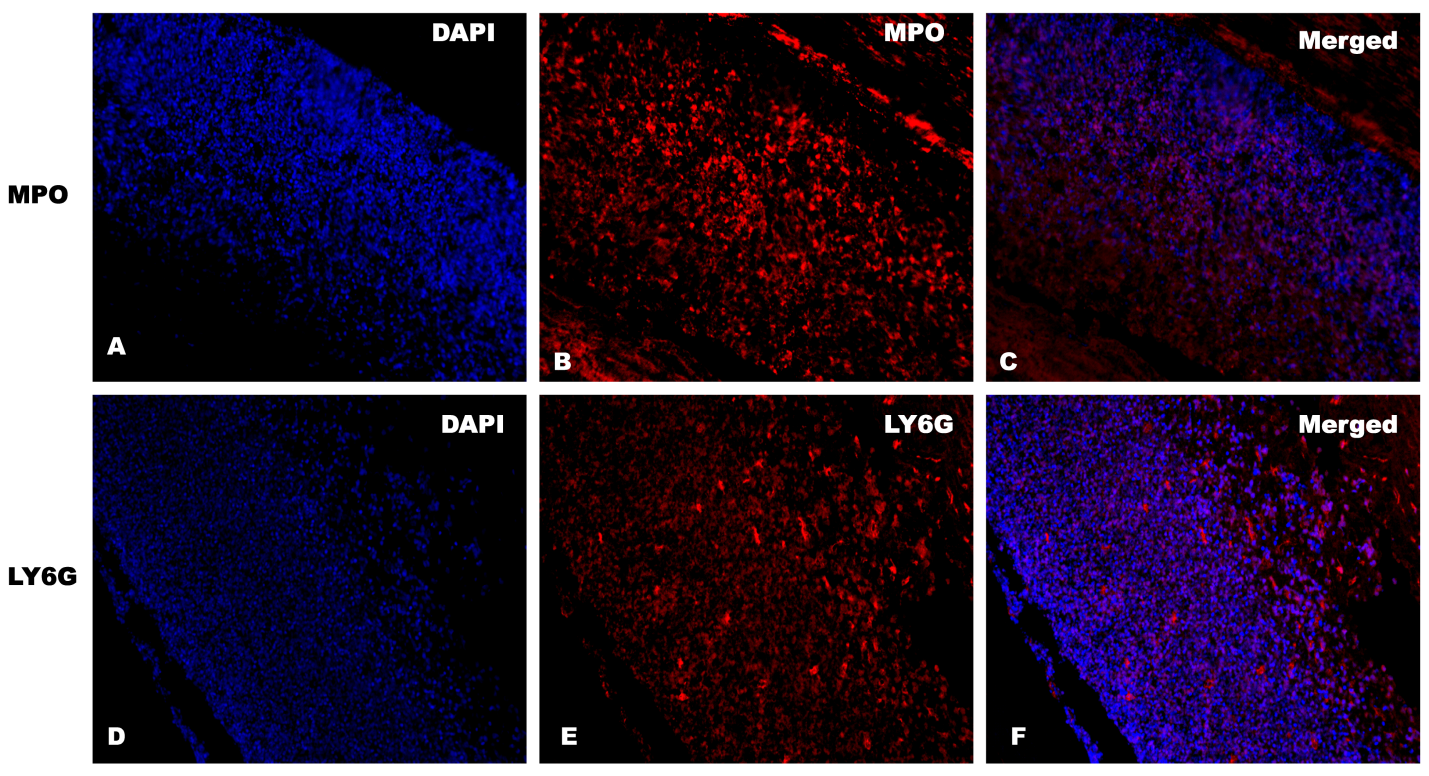
**

Figure S3: Representative immunofluorescence-stained sections for MPO+ and LY6G+ neutrophils of the acute phase graft(red) and DAPI (blue).

1. **Supplemental figure S4: ePTFE graft implantation after 7 years 40.0x**

**
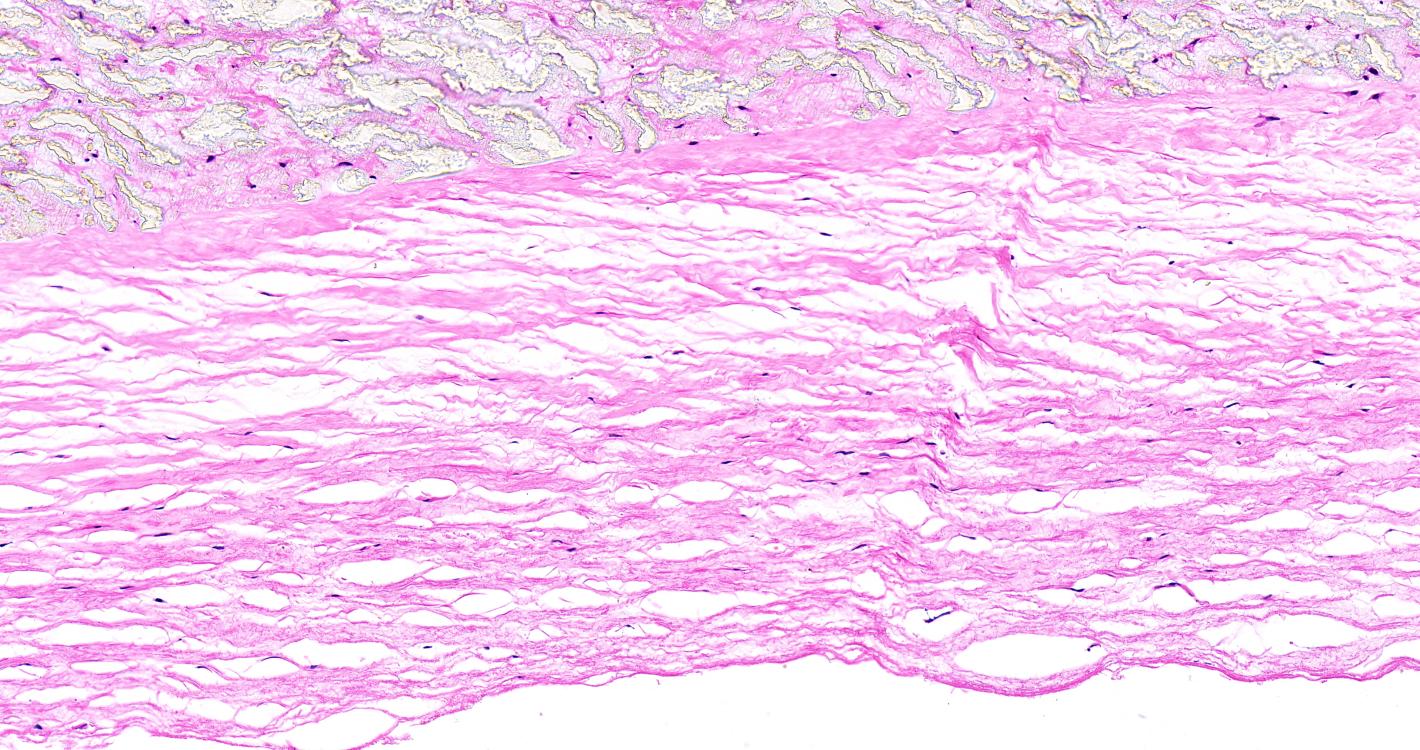
**

Figure S3: HE stain of ePTFE graft explanted during heart transplantation surgery after 7 years in one patient. Image shows the neointima is almost like the native vessel structure with crosslinked collagen network and low-ratio cells infiltration. Magnificant X400.

1. **Potential targets with related expressed proteins (similar to balloon-injury model)**

**Immunofluorescence methods**

Briefly, sections were dewaxed in xylene and rehydrated using a decreasing series of ethanol. Endogenous peroxidase activity was blocked by incubating the slides in 3% methanol. Antigen retrieval was performed by incubating the sections in 0,01M citrate buffer for 30 minutes in a steamer. After cooling the sections to room temperature, a protein block (10% normal goat serum) was used to minimize unspecific binding of the primary antibody. Then follow the steps below.

1. Add primary antibody: Blocking solution was gently shaken off and PBS was added dropwise to the sections with a ratio of well matched primary antibodies (Ly6g, GB11229, 1:500, Servicebio; MPO, GB11224, 1:1000, Servicebio), and the sections were incubated overnight at 4 °C in a flat position inside a wet box. (a small amount of water is added to the wet box to prevent antibody evaporation). The negative control group was added with PBS only.
2. Add secondary antibody: Slides were washed three times for 5 min each in PBS (pH 7.4) with shaking on a destaining shaker. After the sections were slightly wet shaken, the tissues were covered by secondary antibodies (GB21303, 1:300, Servicebio) with the corresponding species of primary antibodies dropwise in the circle and incubated for 50 min in the dark at room temperature.

3. DAPI counterstaining nuclei: after the sections were slightly dried, DAPI (G1012, Servicebio) staining solution was added in the circle and incubated at room temperature for 10 minutes.

4. Autofluorescence quenching: after the slices are slightly dried, add autofluorescence quenching agent (G1401, Servicebio) into the circle for 5min and rinse with running water for 10min.

5. Sealing slide: place the slide in PBS (pH7.4), shake and wash it on the decolorization shaking table for 3 times, each time for 5min. After the slices were slightly dried, they were sealed with anti fluorescence quenching sealing agent.

6. Microscopic examination and photographing: the slices are placed under the scanner (upright fluorescence microscope, Nikon Eclipse C1) to collect images.
